# Supplementary figures and images for: Cell cycle-specific loading of condensin I is regulated by the N-terminal tail of its kleisin subunit
Source: eLife. 2022 Dec 13;11:e84694. doi: 10.7554/eLife.84694 (PMC9797191; doi:10.7554/eLife.84694)

Figure 1-figure supplement 1-source data 1

Figure 1-figure supplement 1A and B

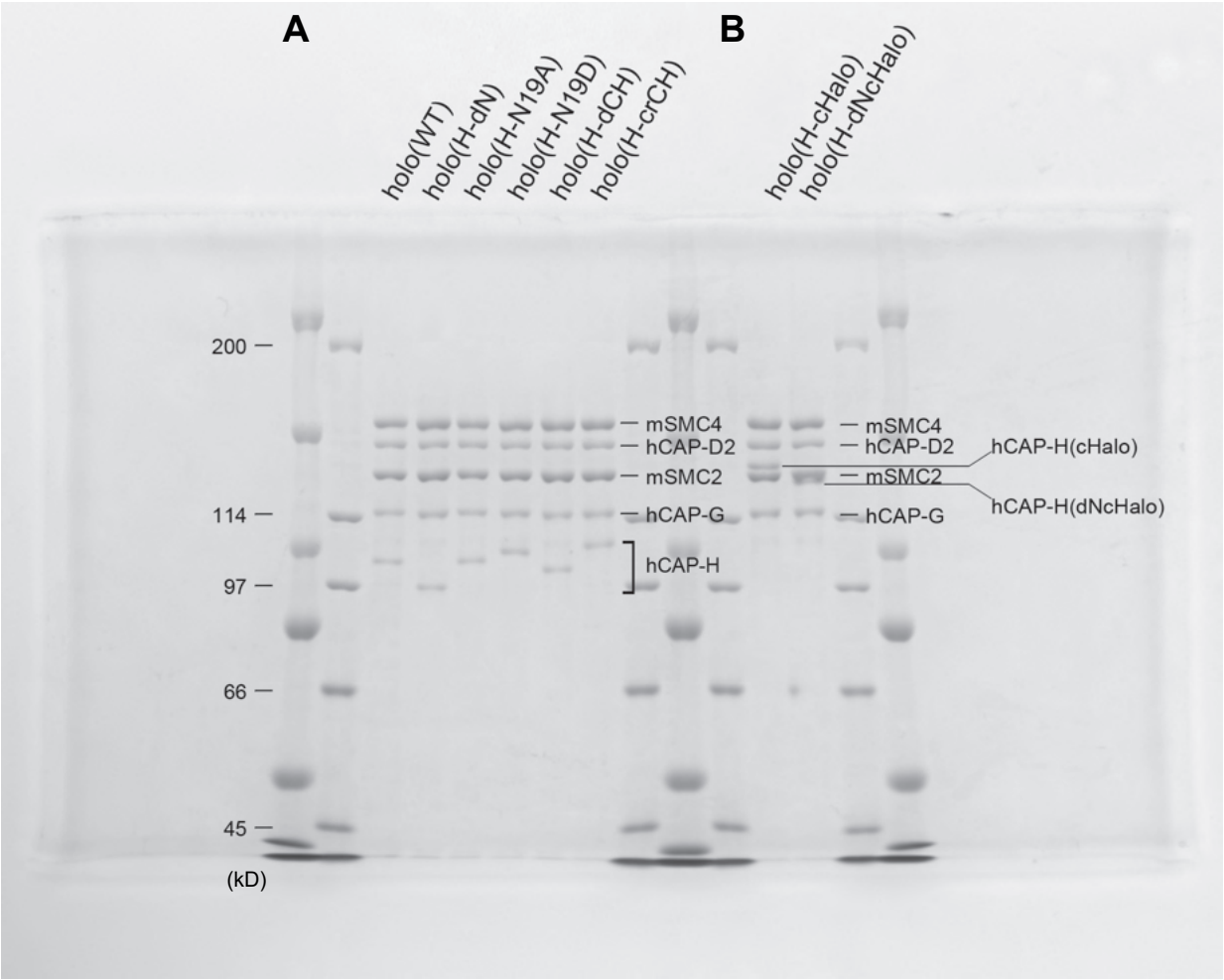

Supplement: Figure 1—figure supplement 1—source data 1. [file elife-84694-fig1-figsupp1-data1.zip › Figure 1-figure supplement 1-source data 1.pdf]

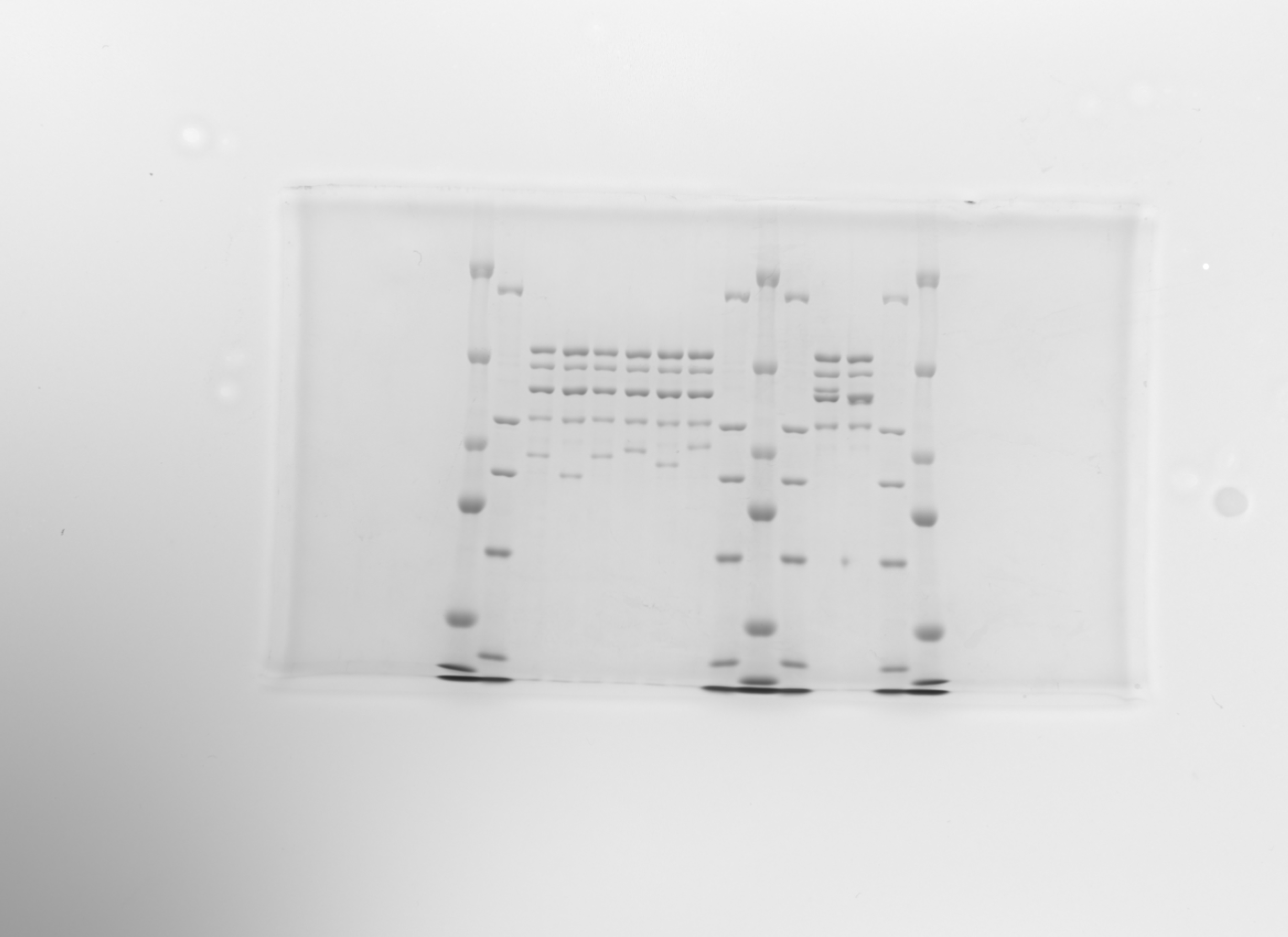

Supplement: Figure 1—figure supplement 1—source data 1. [file elife-84694-fig1-figsupp1-data1.zip › Figure 1-figure supplement 1-source data1.tif]

Figure 1-figure supplement 1-source data 3  
Figure 1-figure supplement 1C

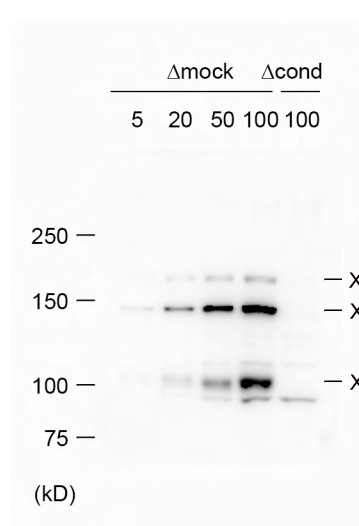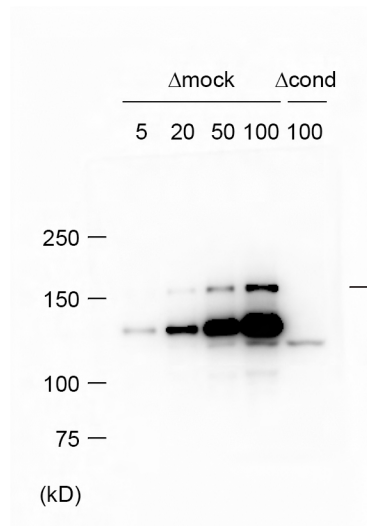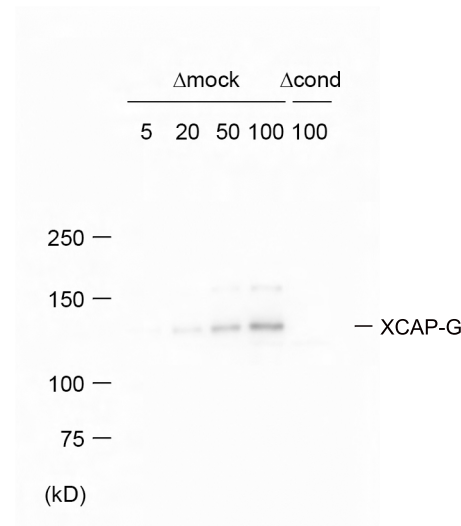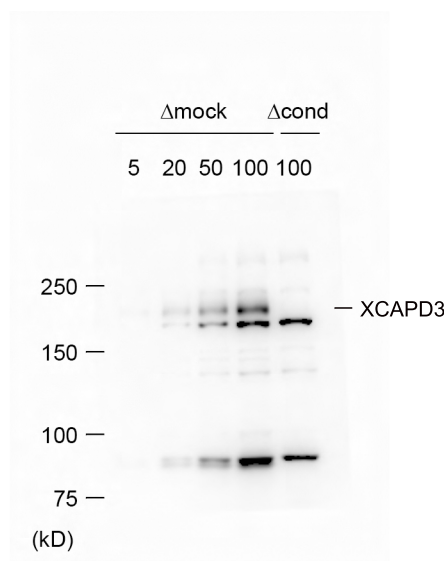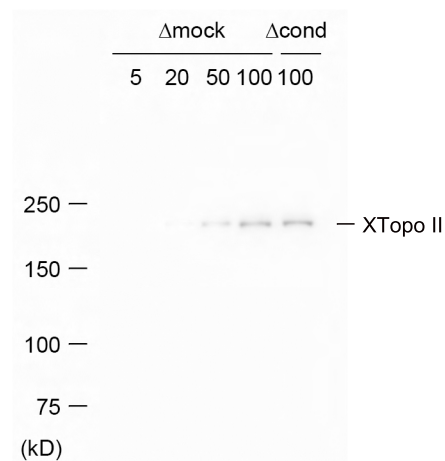

Supplement: Figure 1—figure supplement 1—source data 3. [file elife-84694-fig1-figsupp1-data3.zip › Figure 1-figure supplement 1-source data 3.pdf]

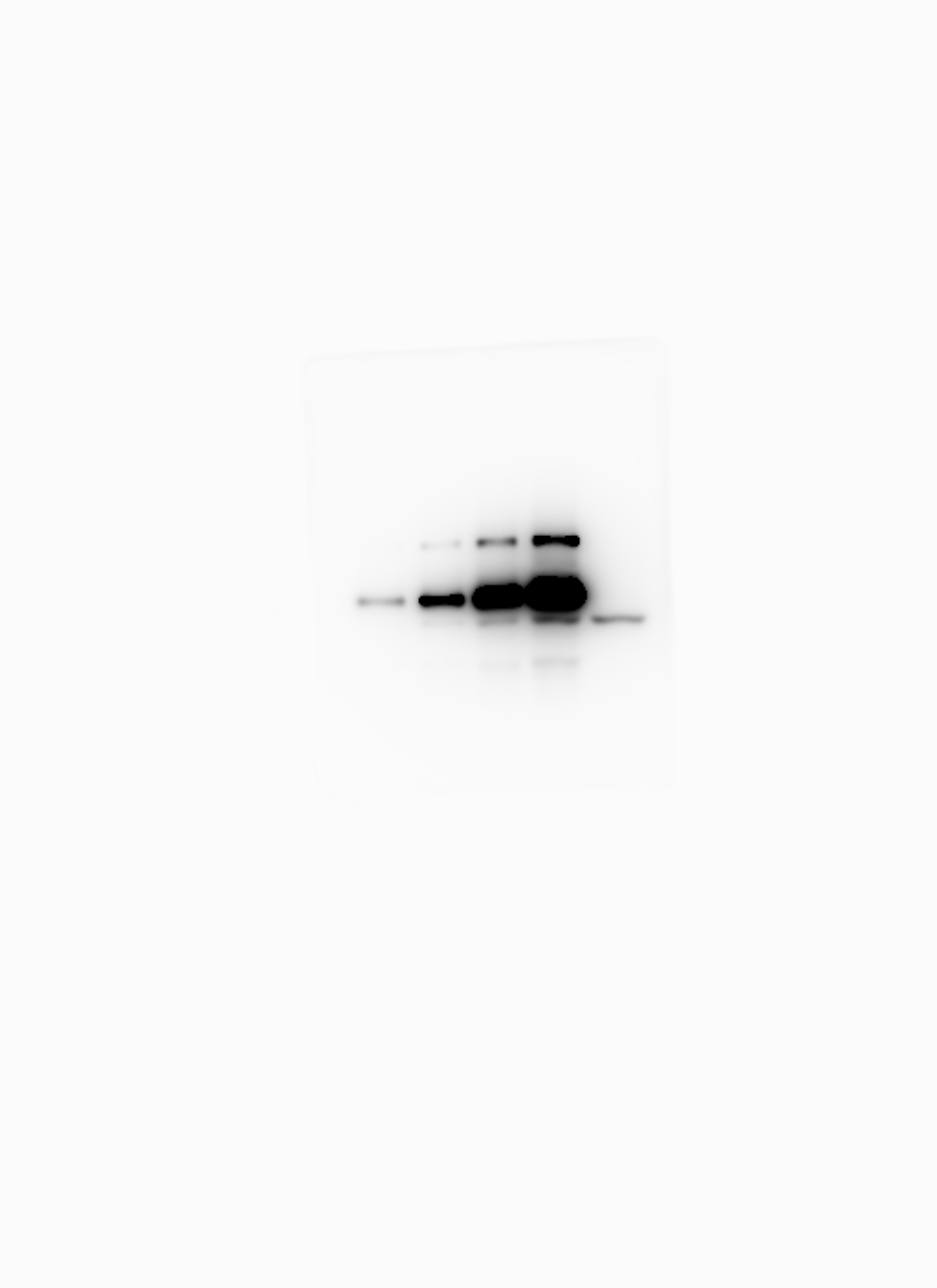

Supplement: Figure 1—figure supplement 1—source data 3. [file elife-84694-fig1-figsupp1-data3.zip › Figure 1-figure supplement 1-source data 3-XCAP-D2.tif]

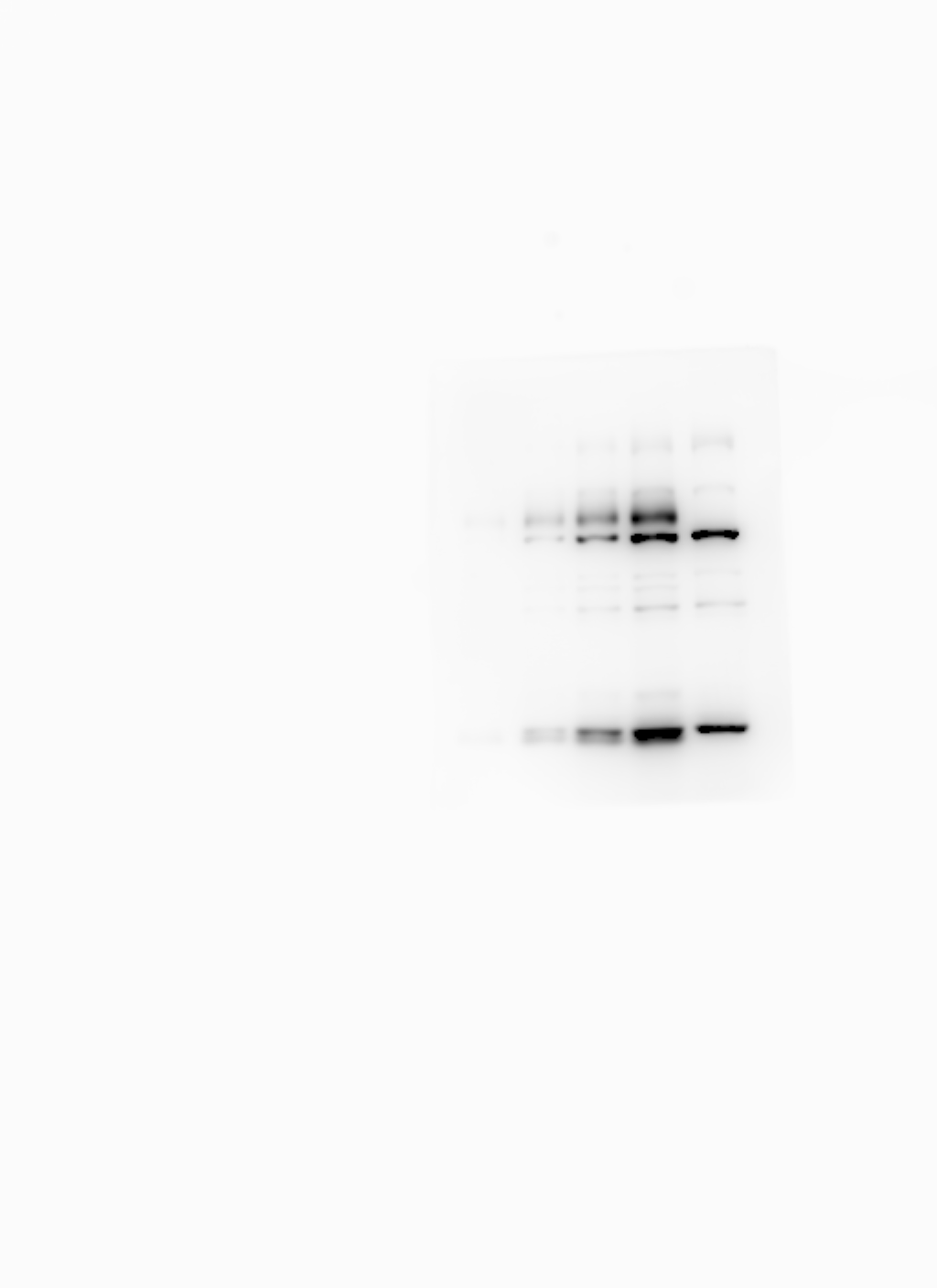

Supplement: Figure 1—figure supplement 1—source data 3. [file elife-84694-fig1-figsupp1-data3.zip › Figure 1-figure supplement 1-source data 3-XCAP-D3.tif]

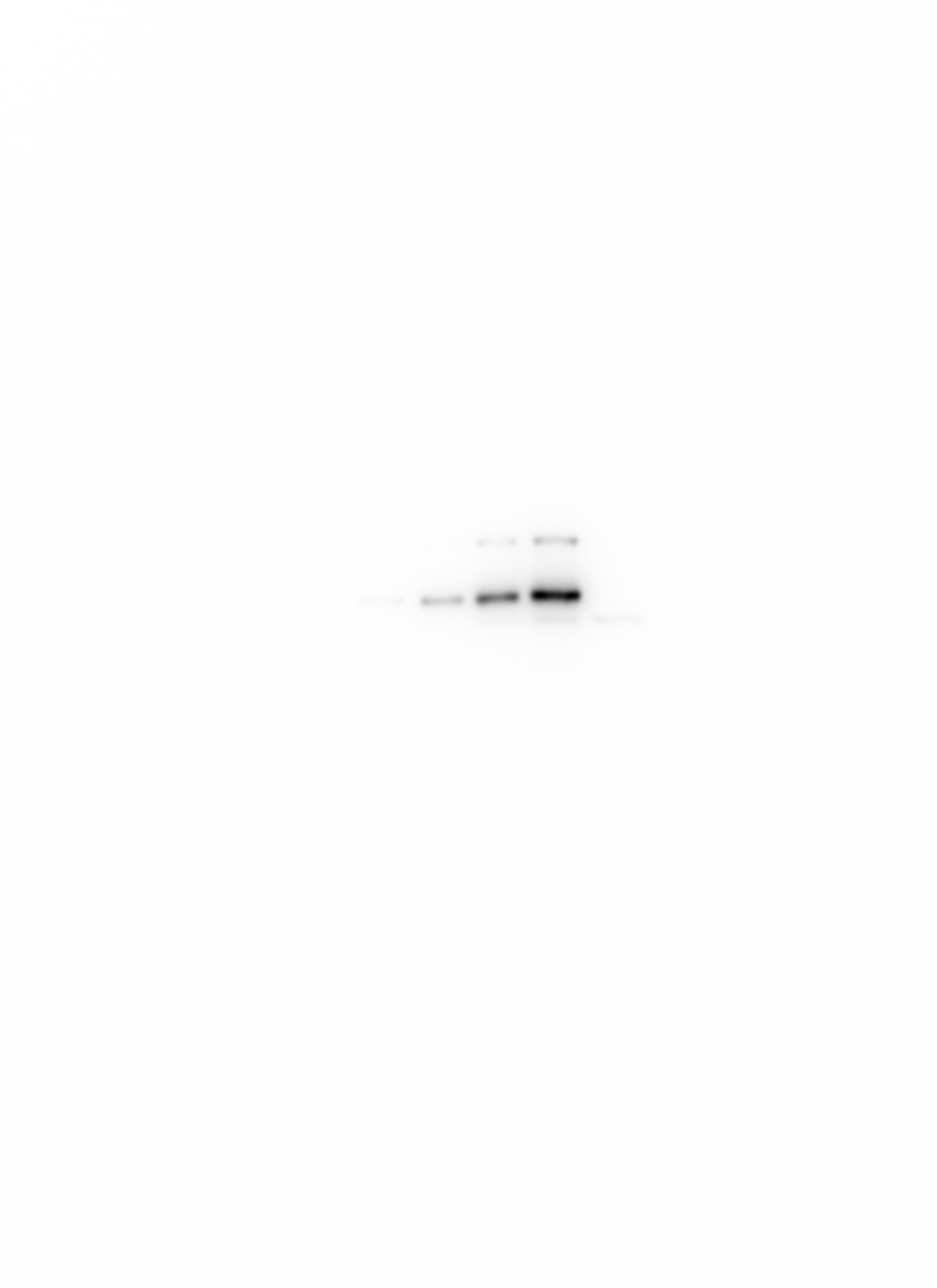

Supplement: Figure 1—figure supplement 1—source data 3. [file elife-84694-fig1-figsupp1-data3.zip › Figure 1-figure supplement 1-source data 3-XCAP-G.tif]

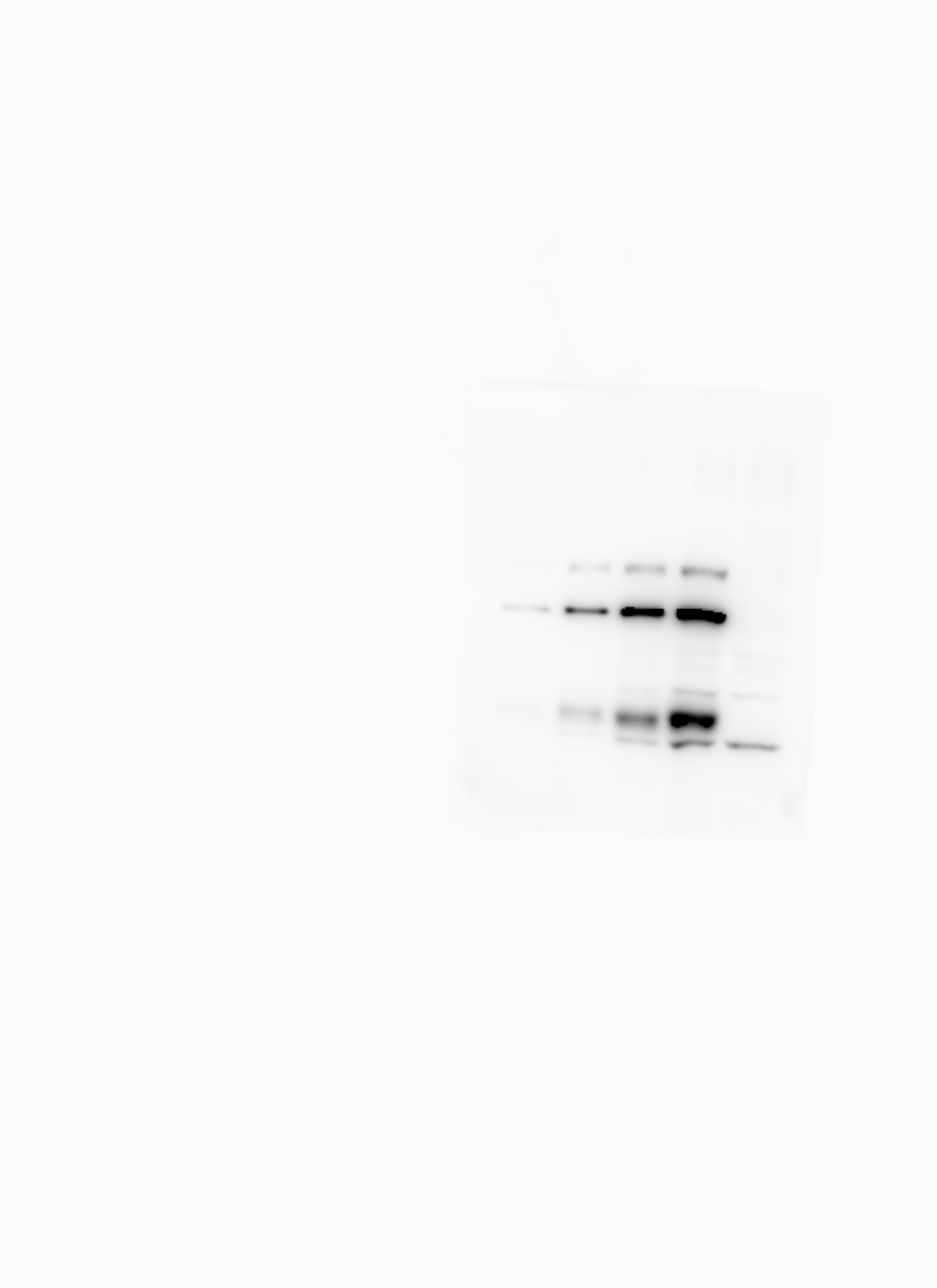

Supplement: Figure 1—figure supplement 1—source data 3. [file elife-84694-fig1-figsupp1-data3.zip › Figure 1-figure supplement 1-source data 3-XSMC2, XSMC4 and XCAP-H.tif]

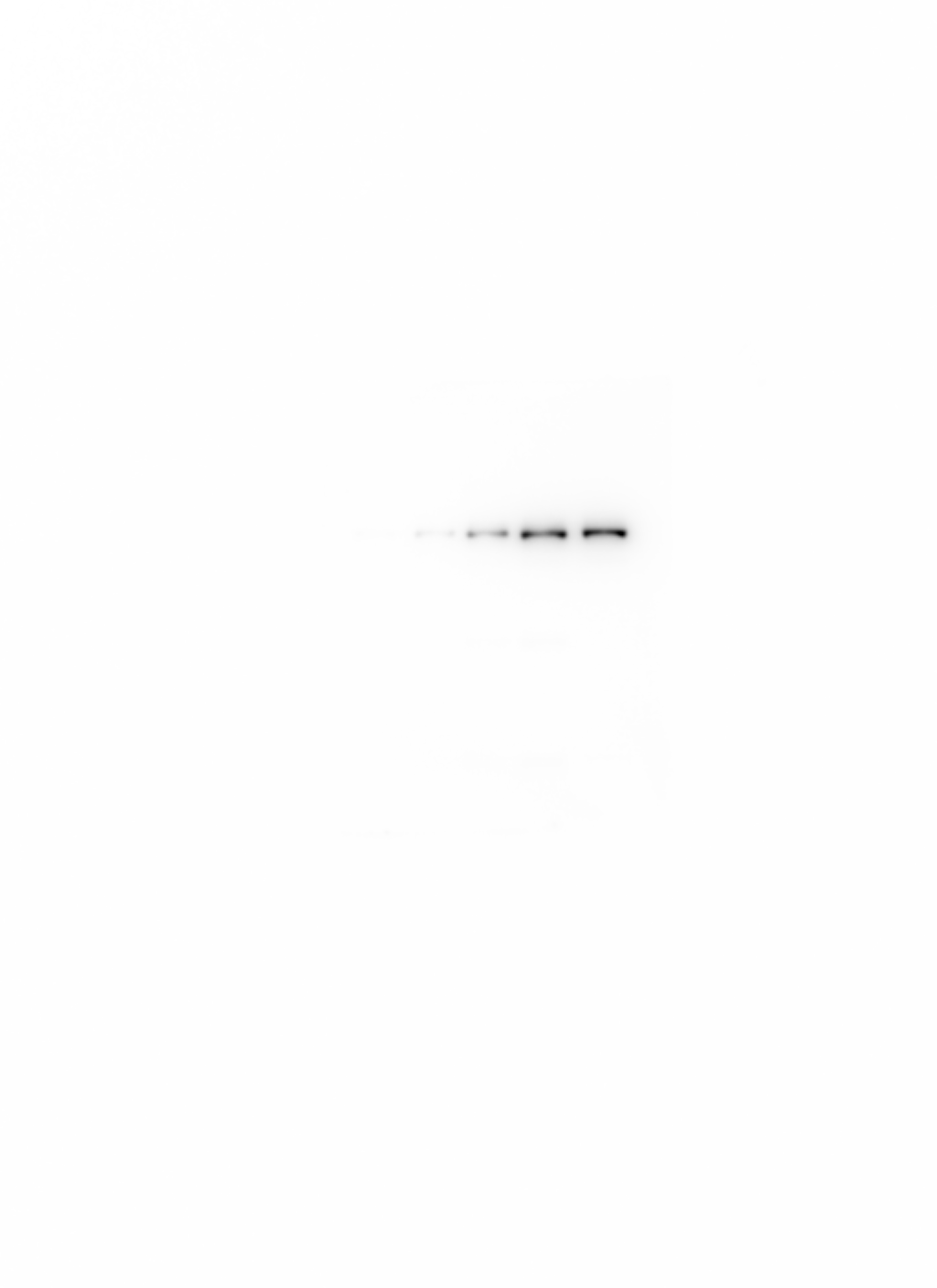

Supplement: Figure 1—figure supplement 1—source data 3. [file elife-84694-fig1-figsupp1-data3.zip › Figure 1-figure supplement 1-source data 3-XTopo II.tif]

Figure 2-figure supplement 1-source data 1  
Figure 2-figure supplement 1B

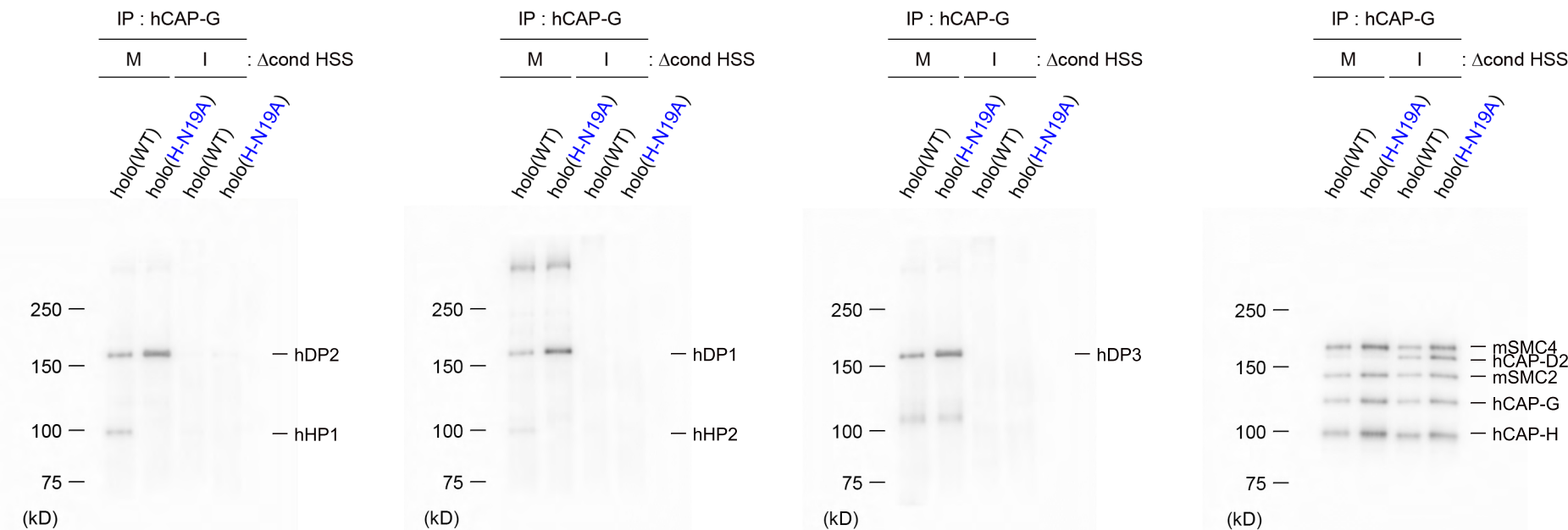

Supplement: Figure 2—figure supplement 1—source data 1. [file elife-84694-fig2-figsupp1-data1.zip › Figure 2-figure supplement 1-source data 1.pdf]

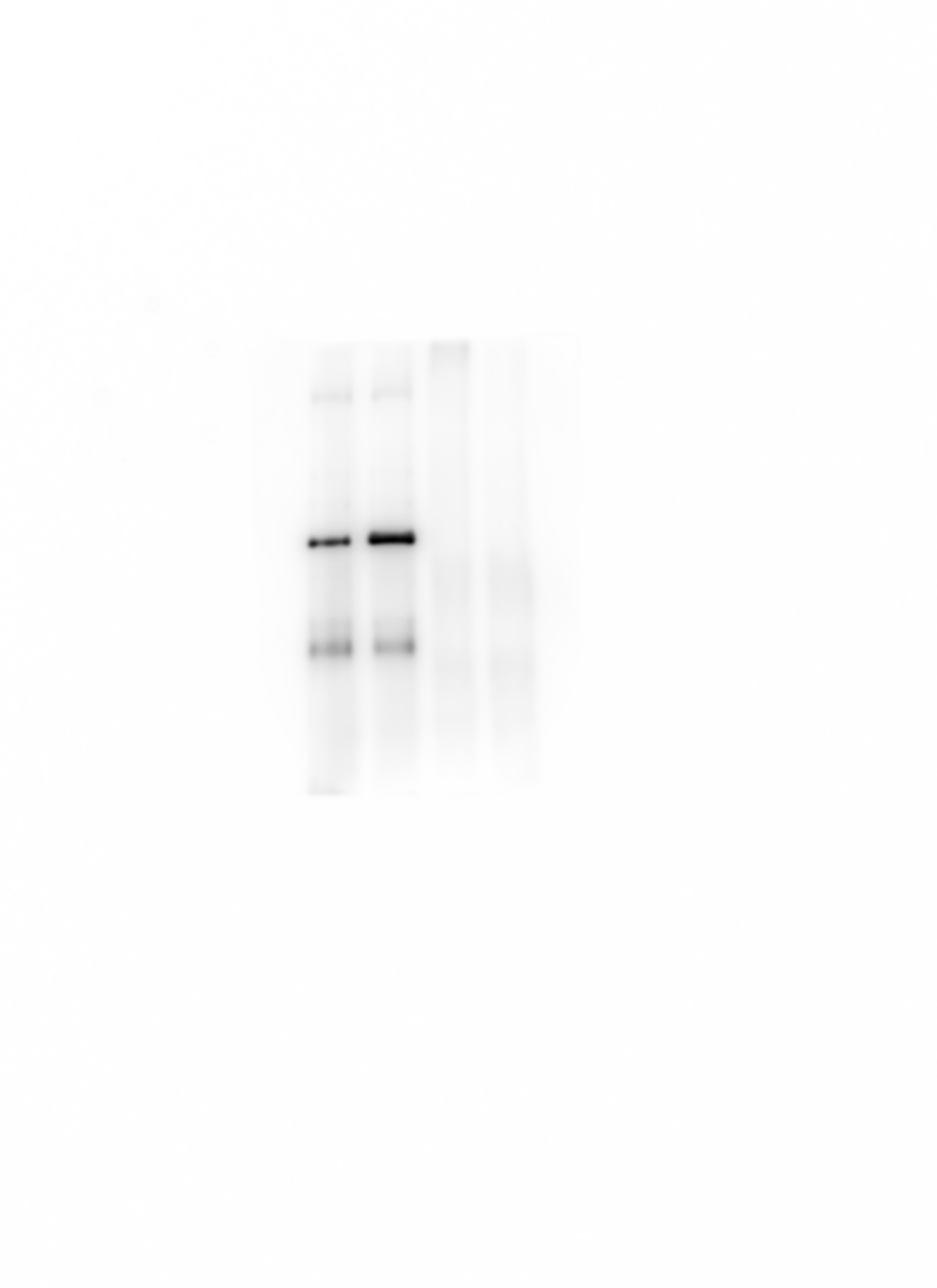

Supplement: Figure 2—figure supplement 1—source data 1. [file elife-84694-fig2-figsupp1-data1.zip › Figure 2-figure supplement 1-source data 1-hDP3.tif]

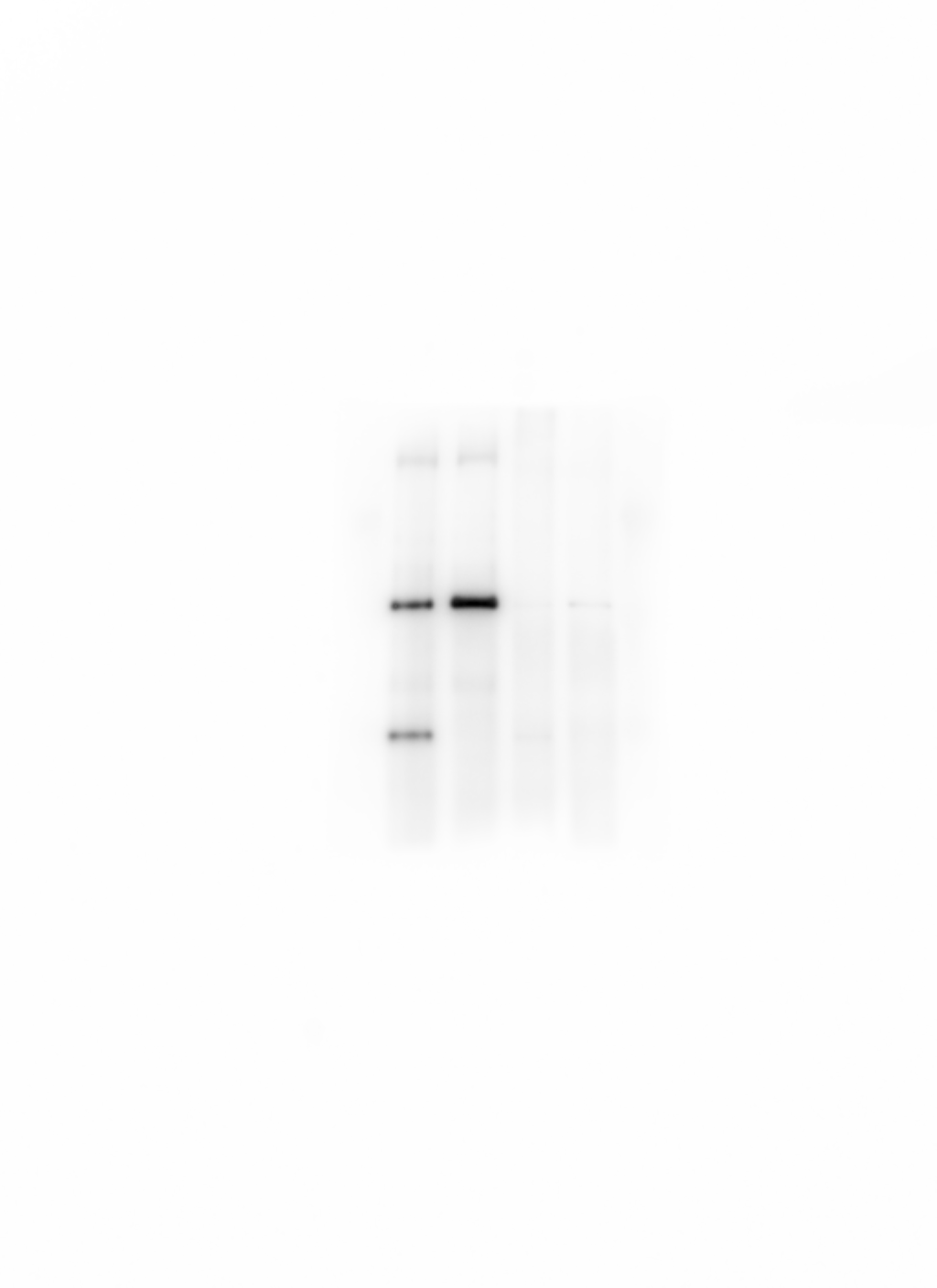

Supplement: Figure 2—figure supplement 1—source data 1. [file elife-84694-fig2-figsupp1-data1.zip › Figure 2-figure supplement 1-source data 1-hHP1 and hDP2.tif]

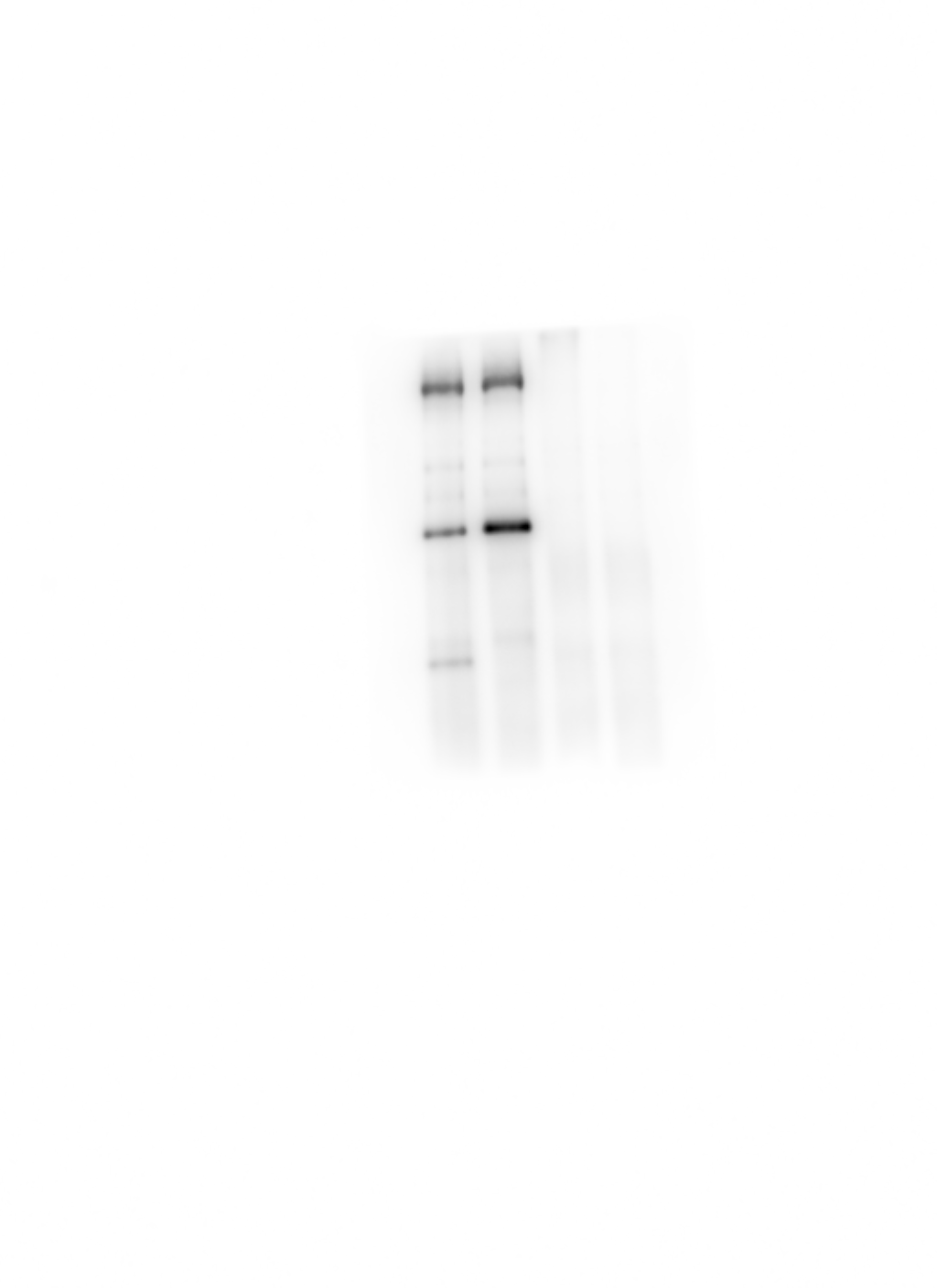

Supplement: Figure 2—figure supplement 1—source data 1. [file elife-84694-fig2-figsupp1-data1.zip › Figure 2-figure supplement 1-source data 1-hHP2 and hDP1.tif]

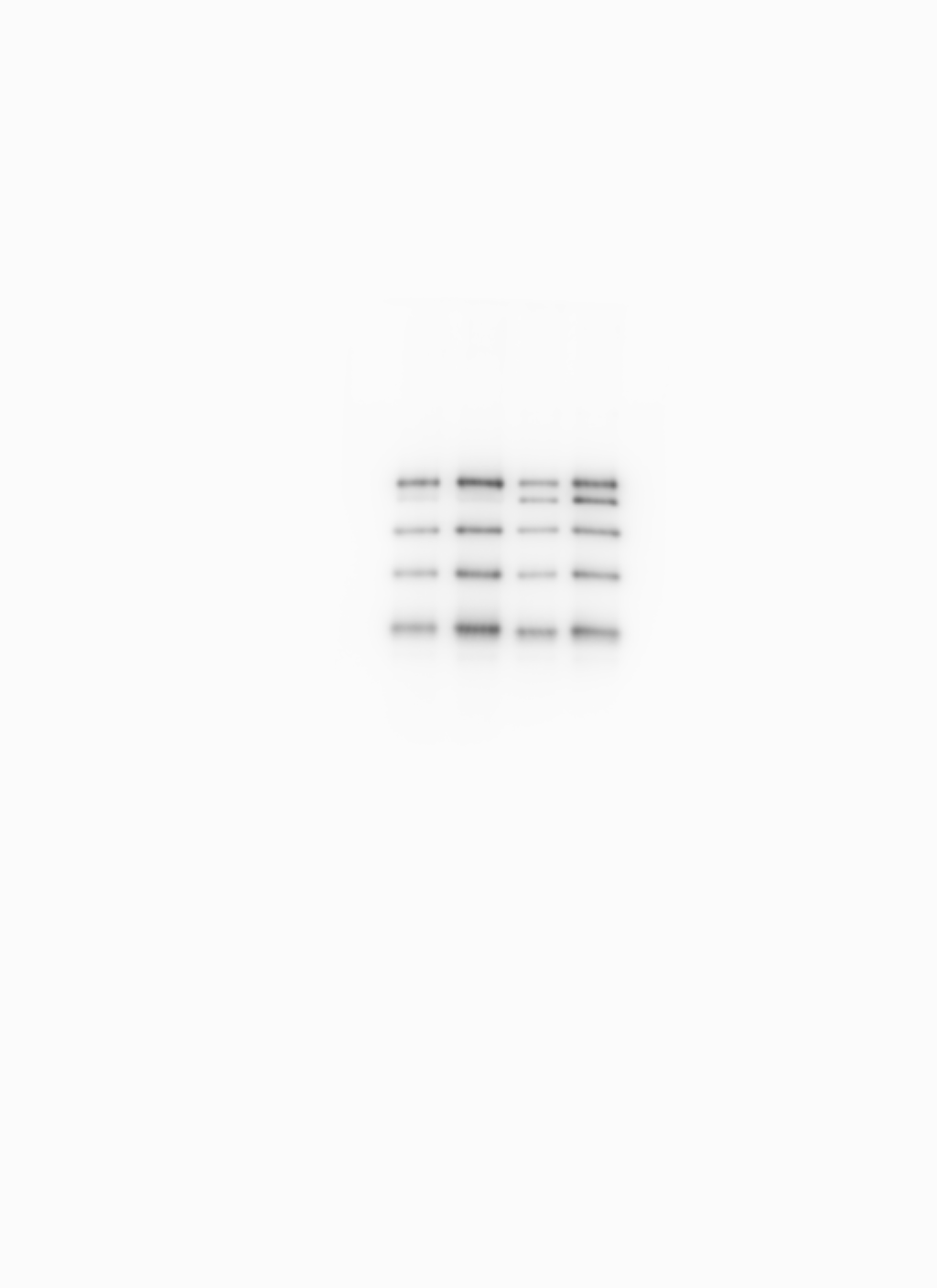

Supplement: Figure 2—figure supplement 1—source data 1. [file elife-84694-fig2-figsupp1-data1.zip › Figure 2-figure supplement 1-source data 1-holo(WT) and holo(H-N19A).tif]

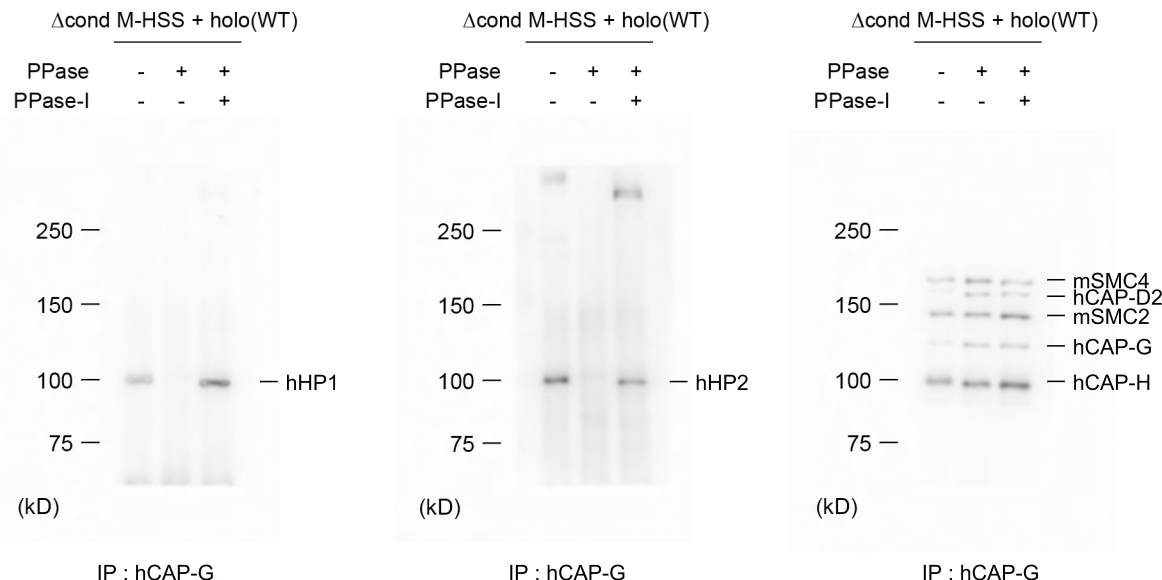

Supplement: Figure 2—figure supplement 1—source data 2. [file elife-84694-fig2-figsupp1-data2.zip › Figure 2-figure supplement 1-source data 2.pdf]

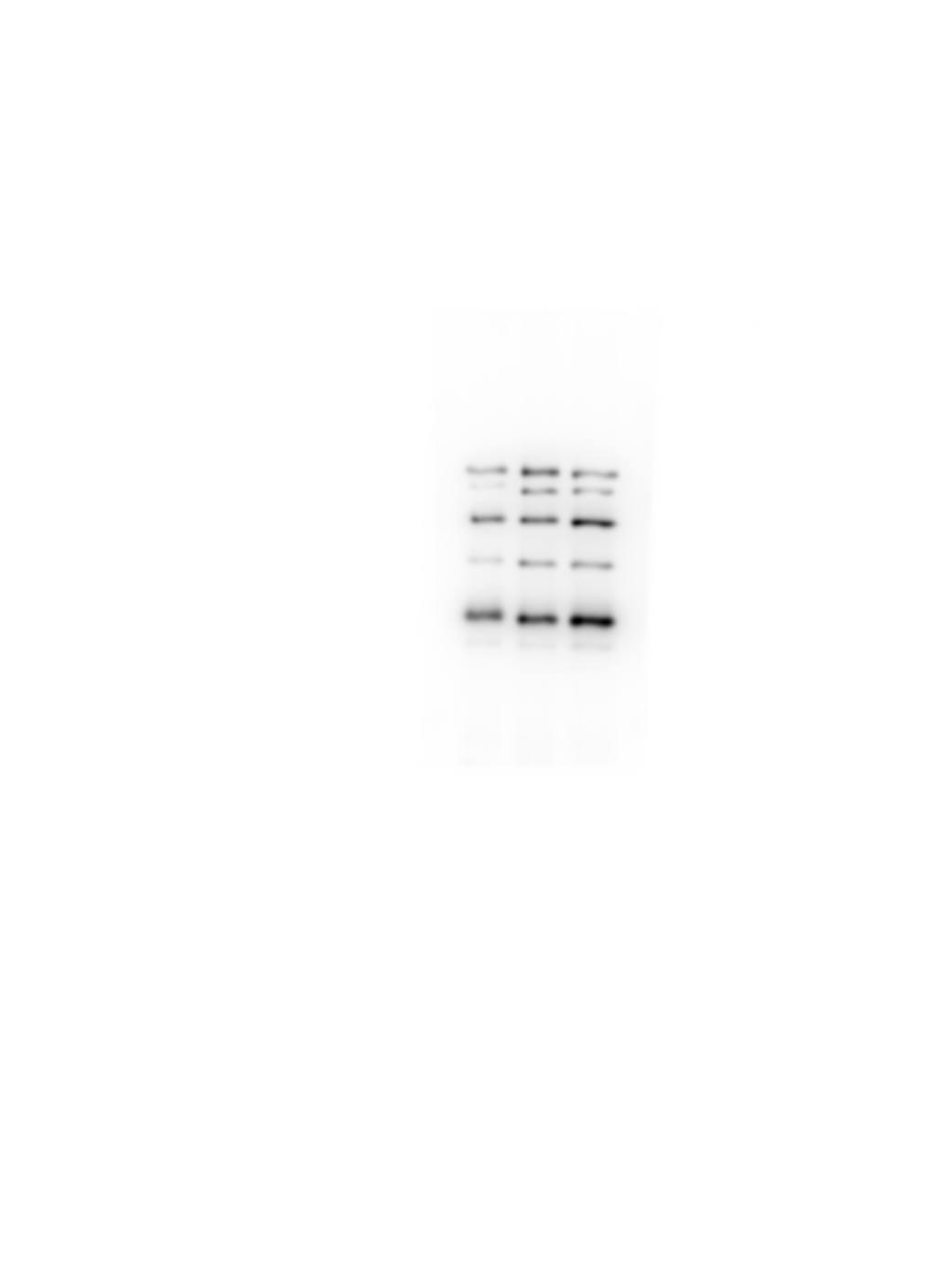

Supplement: Figure 2—figure supplement 1—source data 2. [file elife-84694-fig2-figsupp1-data2.zip › Figure 2-figure supplement 1-source data 2-hCAP-H.tif]

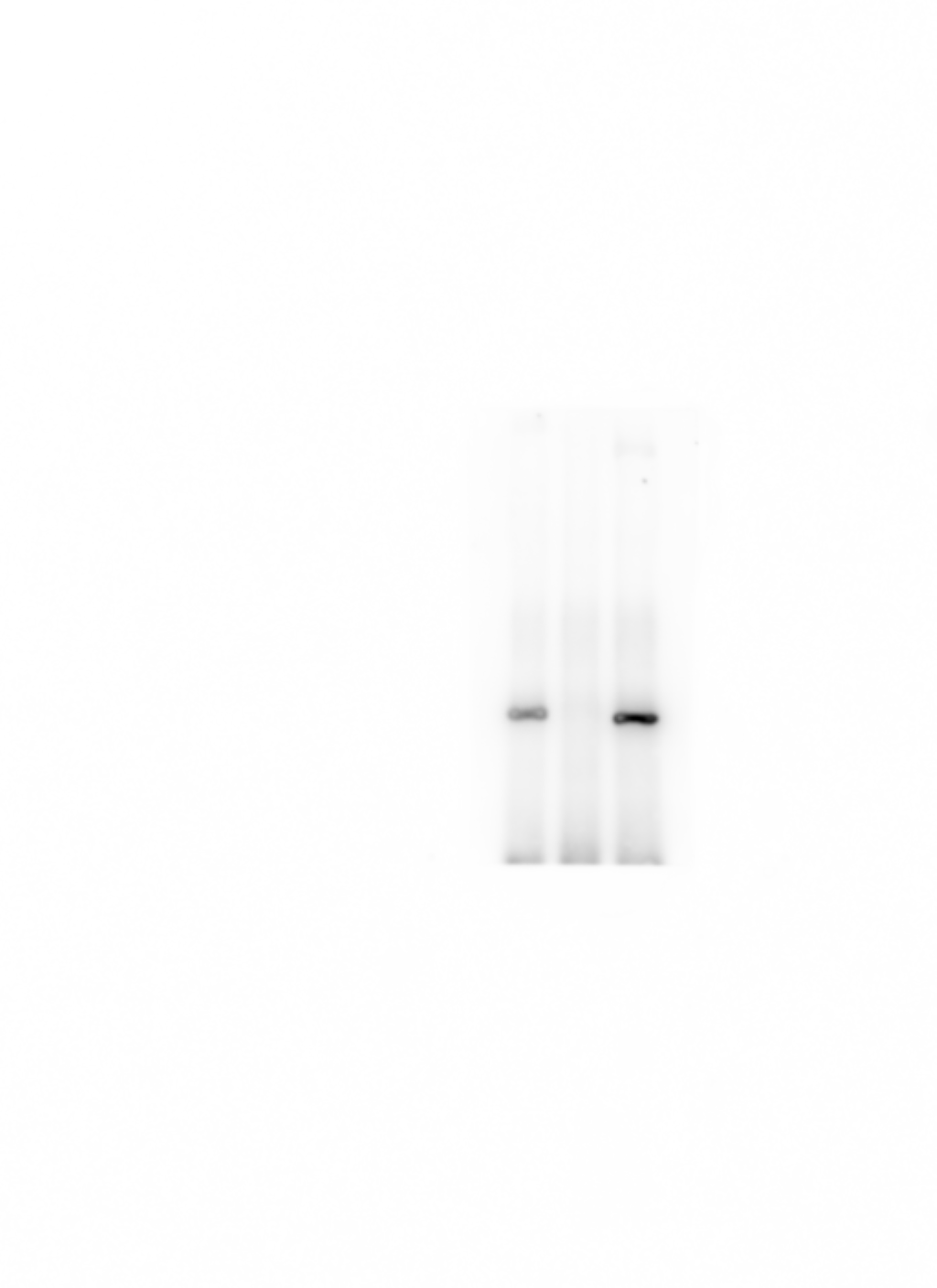

Supplement: Figure 2—figure supplement 1—source data 2. [file elife-84694-fig2-figsupp1-data2.zip › Figure 2-figure supplement 1-source data 2-hHP1.tif]

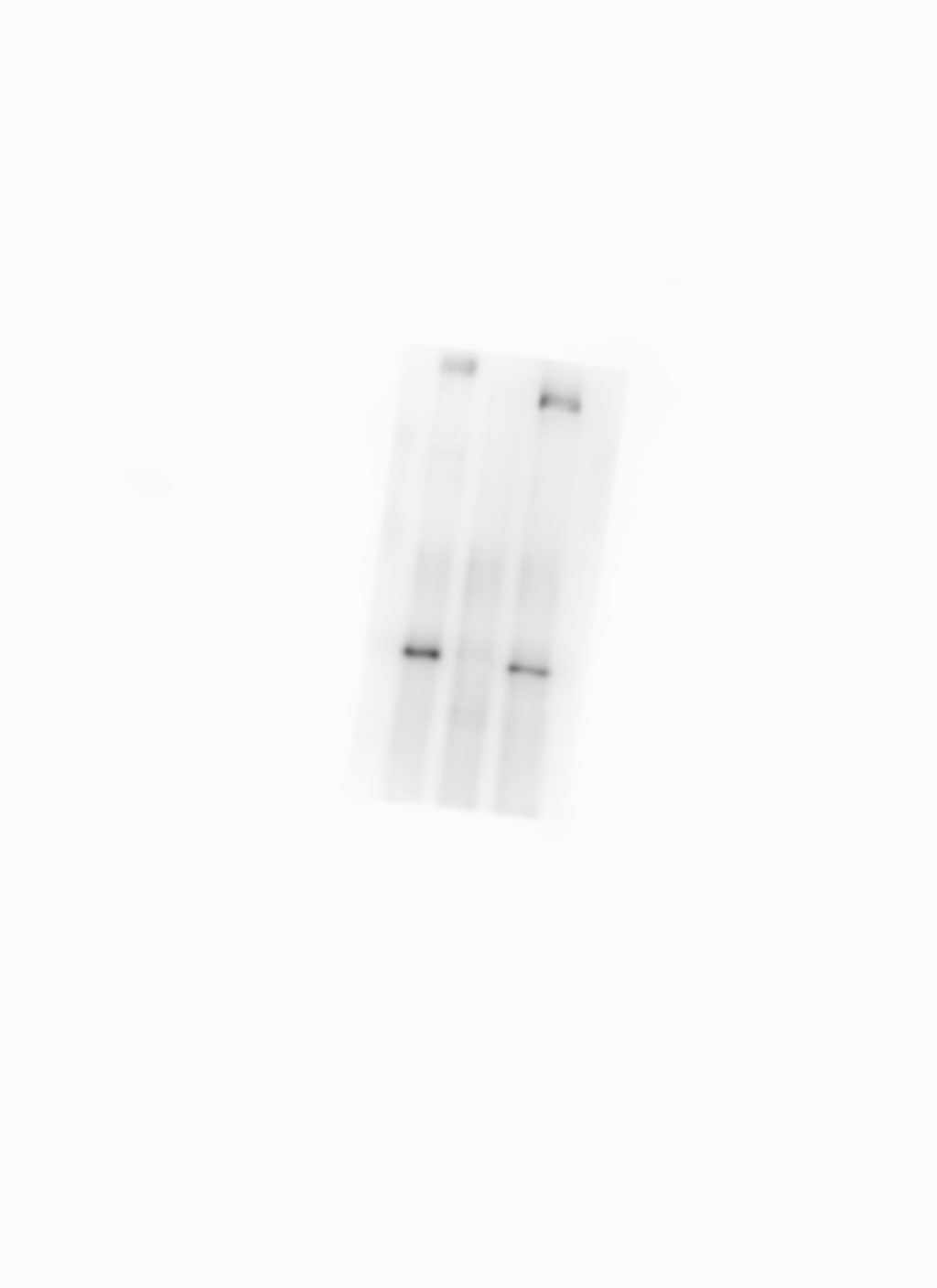

Supplement: Figure 2—figure supplement 1—source data 2. [file elife-84694-fig2-figsupp1-data2.zip › Figure 2-figure supplement 1-source data 2-hHP2.tif]

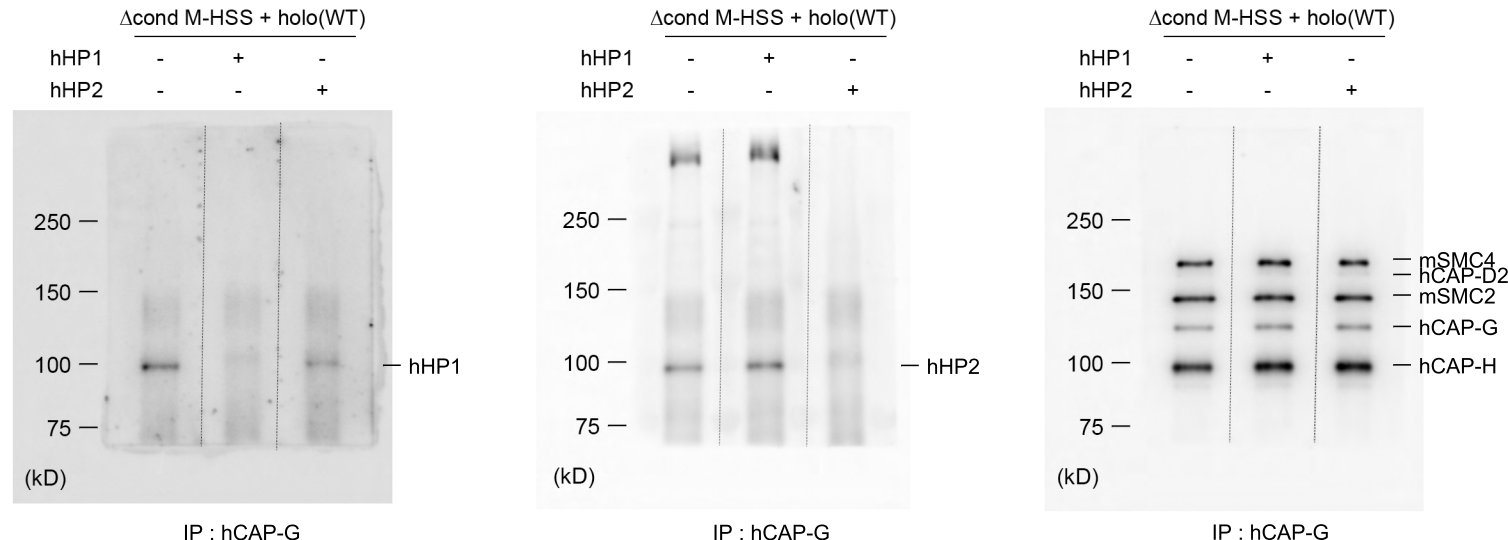

Supplement: Figure 2—figure supplement 1—source data 3. [file elife-84694-fig2-figsupp1-data3.zip › Figure 2-figure supplement 1-source data 3.pdf]

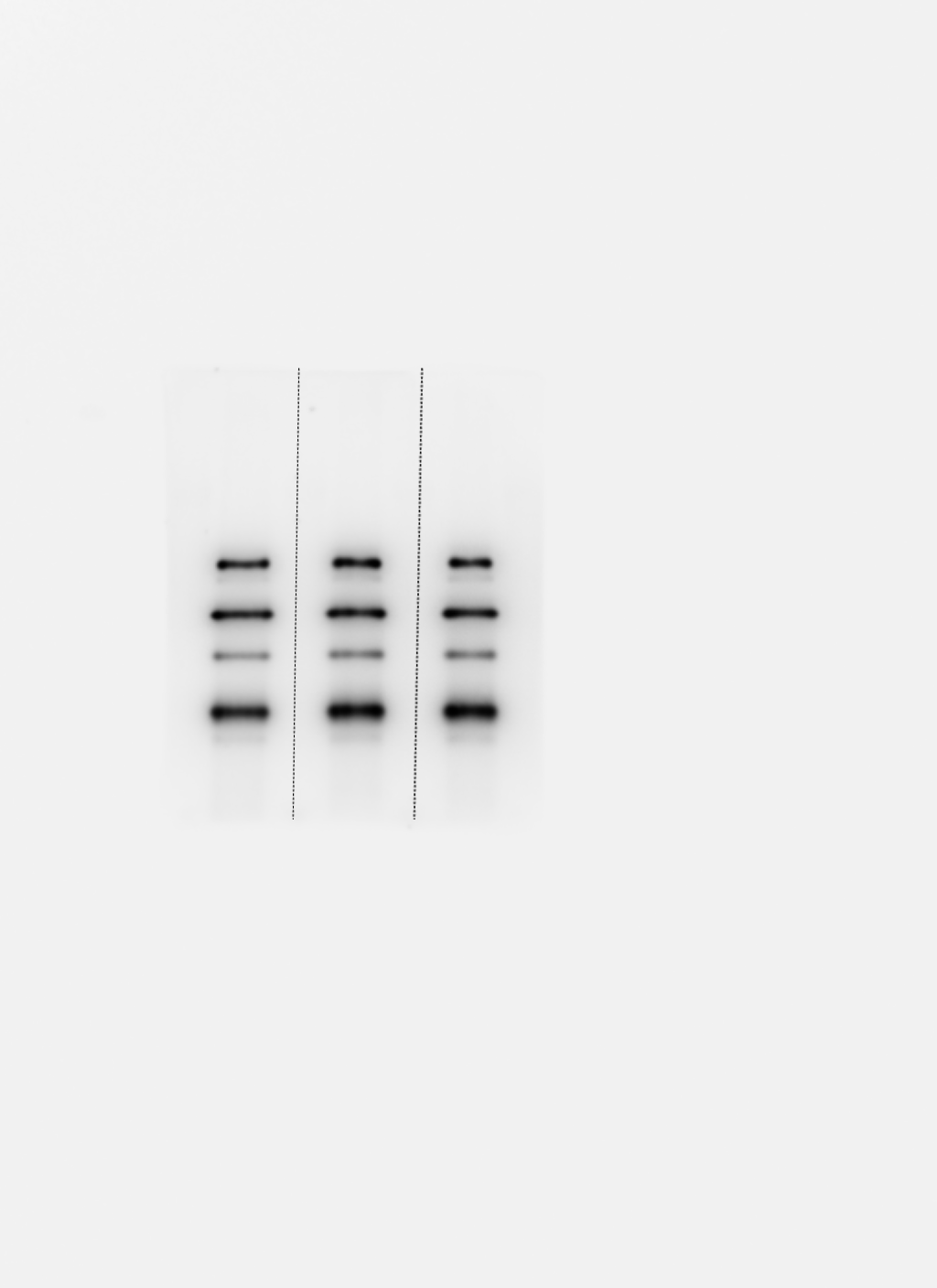

Supplement: Figure 2—figure supplement 1—source data 3. [file elife-84694-fig2-figsupp1-data3.zip › Figure 2-figure supplement 1-source data 3-hCAP-H.tif]

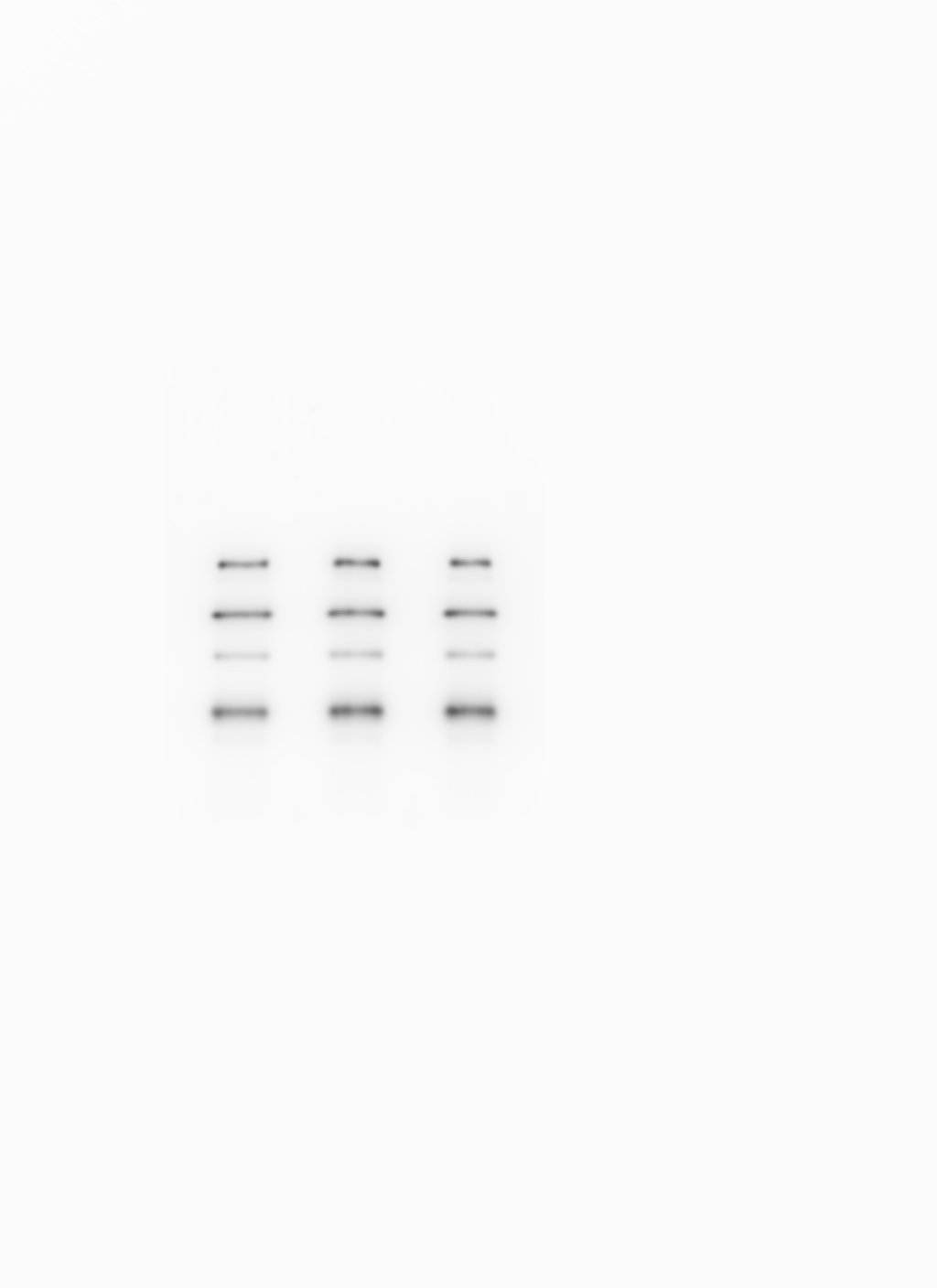

Supplement: Figure 2—figure supplement 1—source data 3. [file elife-84694-fig2-figsupp1-data3.zip › Figure 2-figure supplement 1-source data 3-hCAP-H_original.tif]

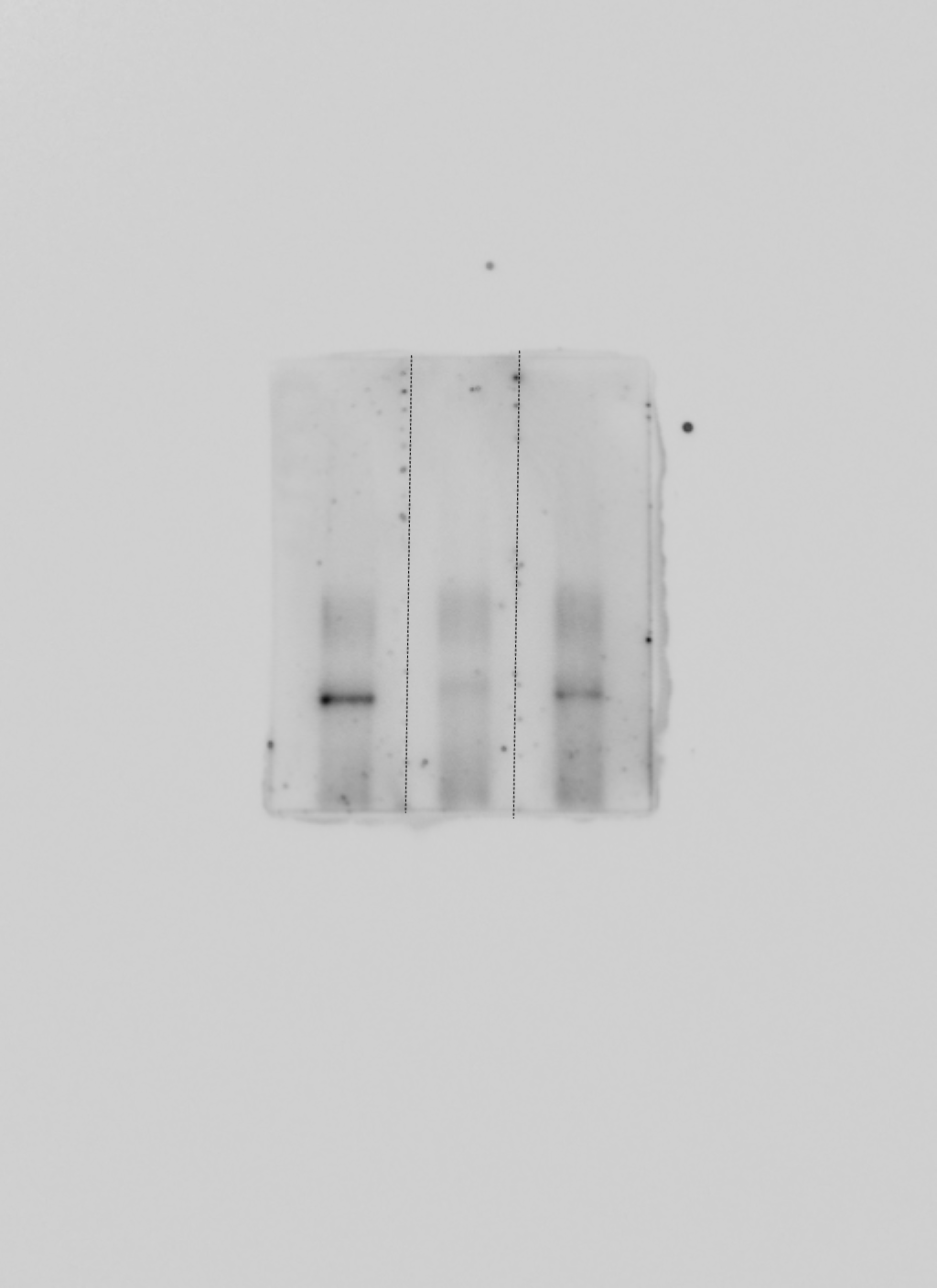

Supplement: Figure 2—figure supplement 1—source data 3. [file elife-84694-fig2-figsupp1-data3.zip › Figure 2-figure supplement 1-source data 3-hHP1.tif]

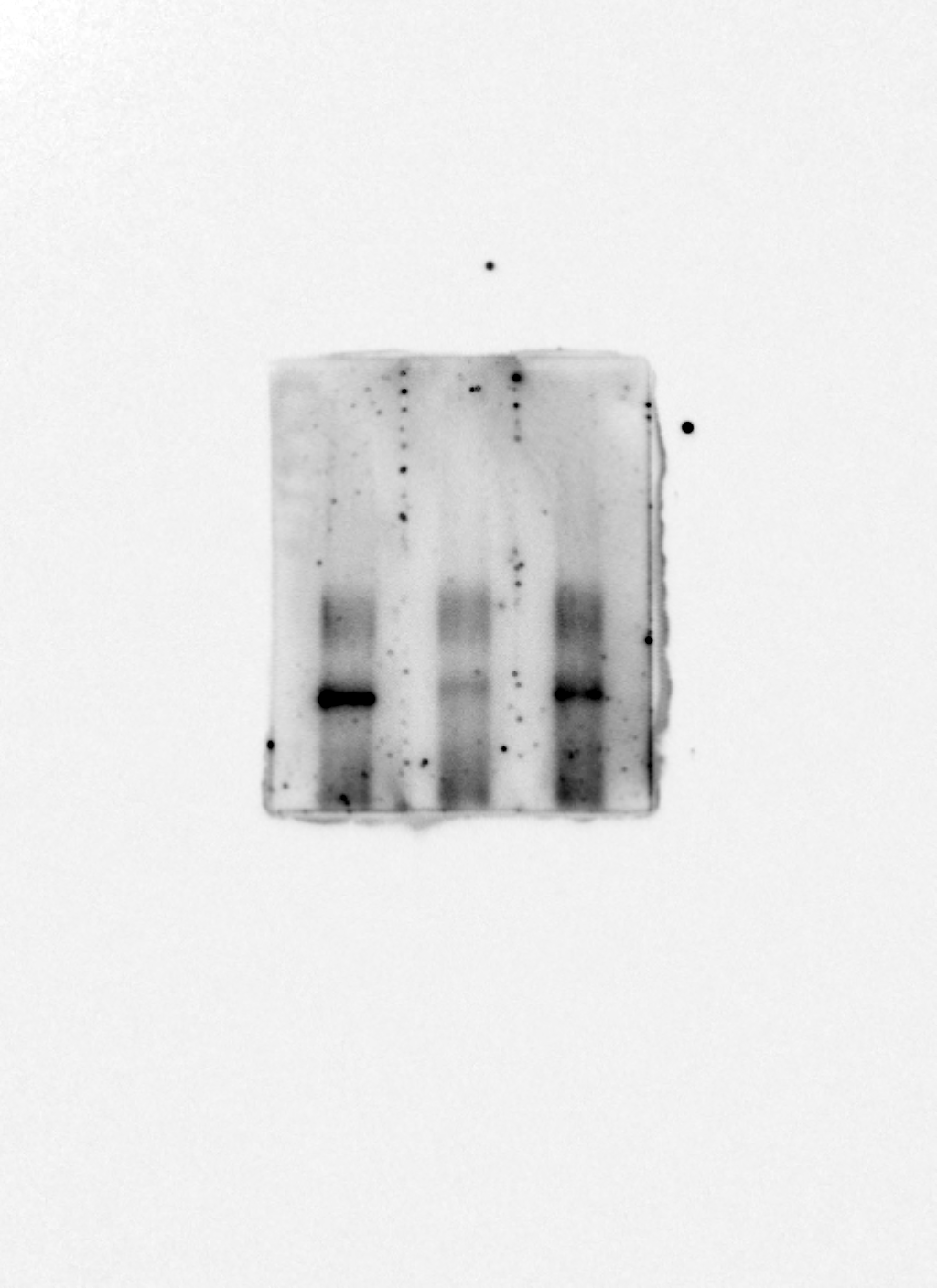

Supplement: Figure 2—figure supplement 1—source data 3. [file elife-84694-fig2-figsupp1-data3.zip › Figure 2-figure supplement 1-source data 3-hHP1_original.tif]

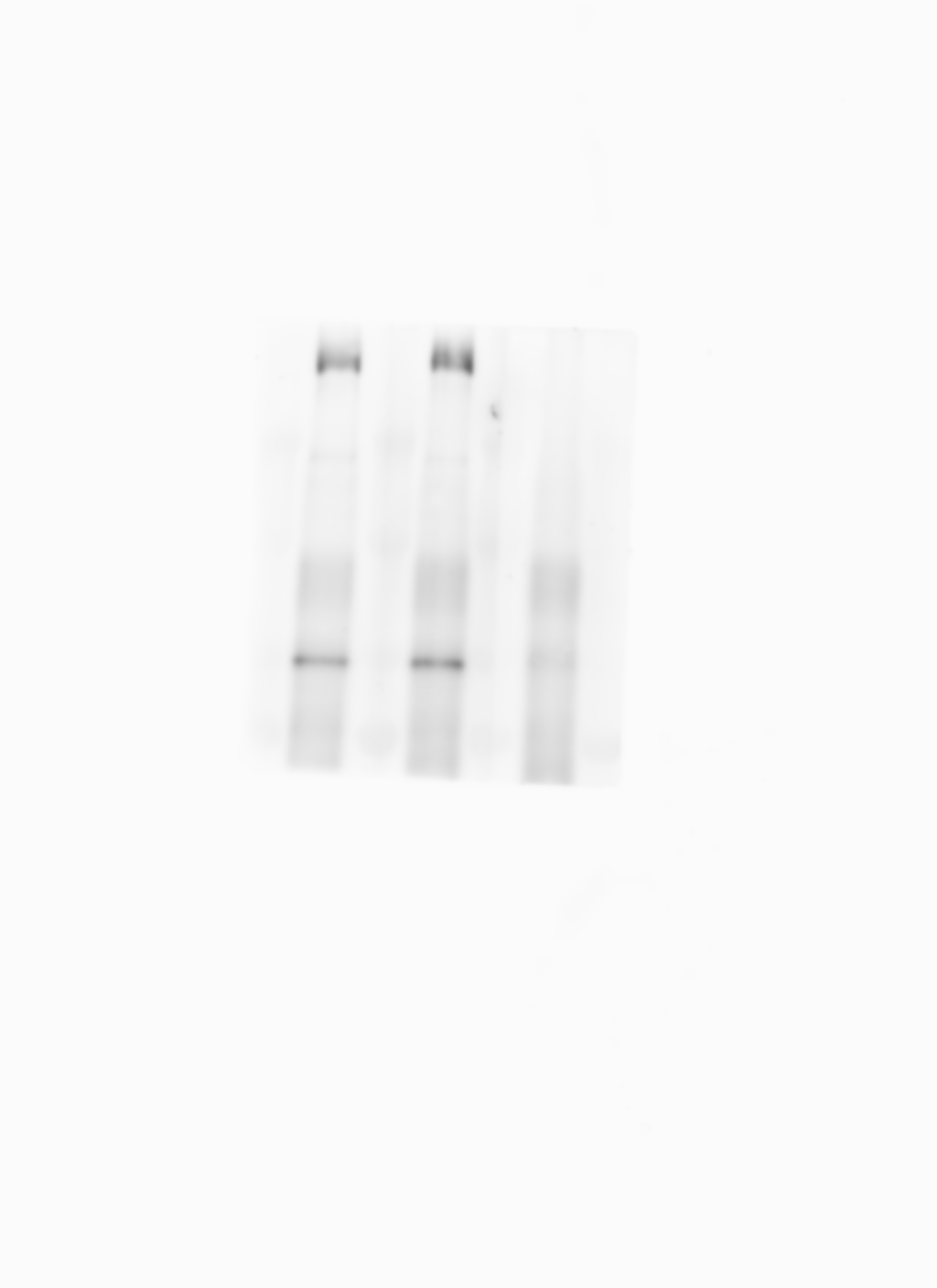

Supplement: Figure 2—figure supplement 1—source data 3. [file elife-84694-fig2-figsupp1-data3.zip › Figure 2-figure supplement 1-source data 3-hHP2 _original.tif]

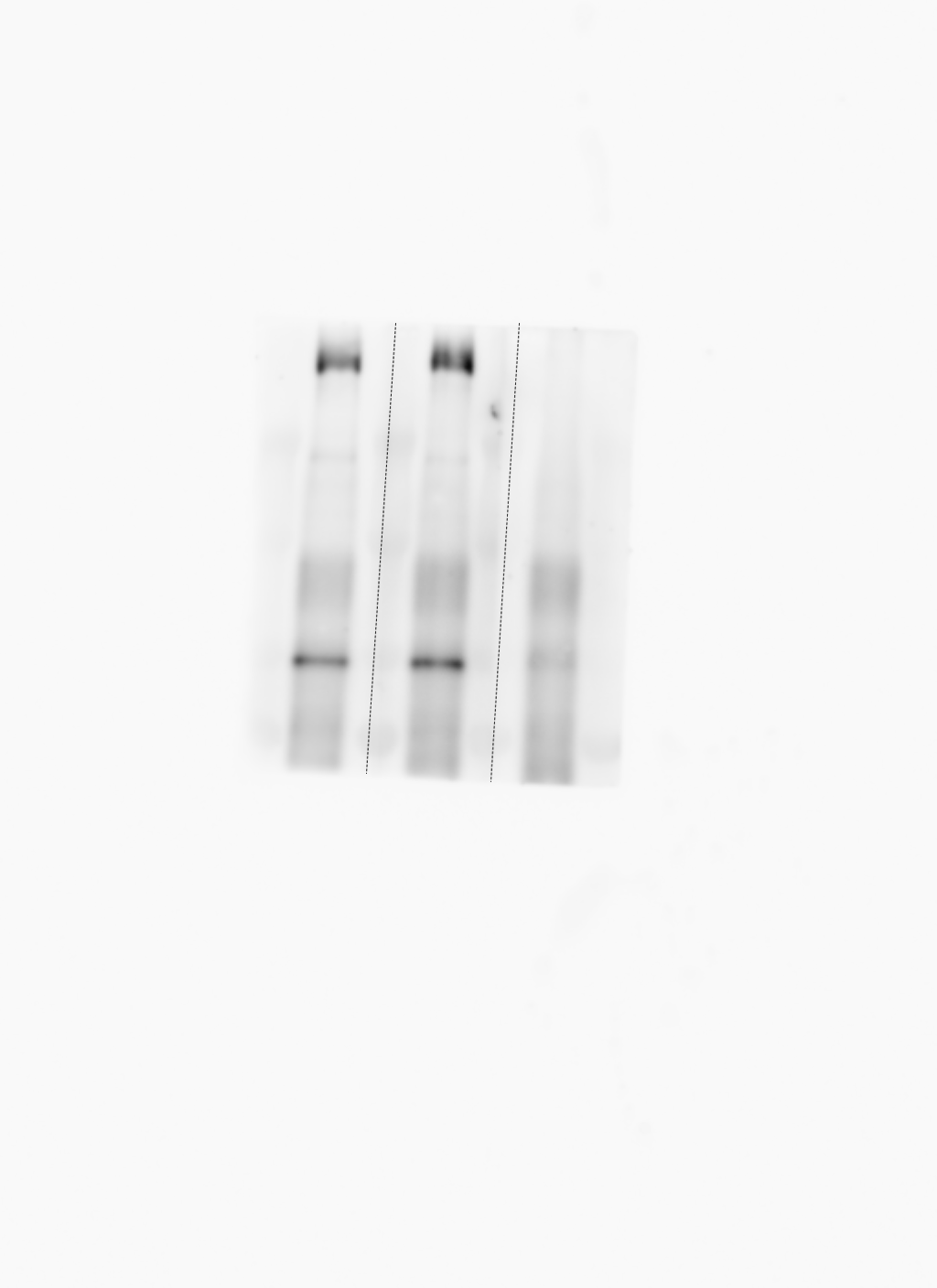

Supplement: Figure 2—figure supplement 1—source data 3. [file elife-84694-fig2-figsupp1-data3.zip › Figure 2-figure supplement 1-source data 3-hHP2.tif]

Figure 4-source data 1  
Figure 4A

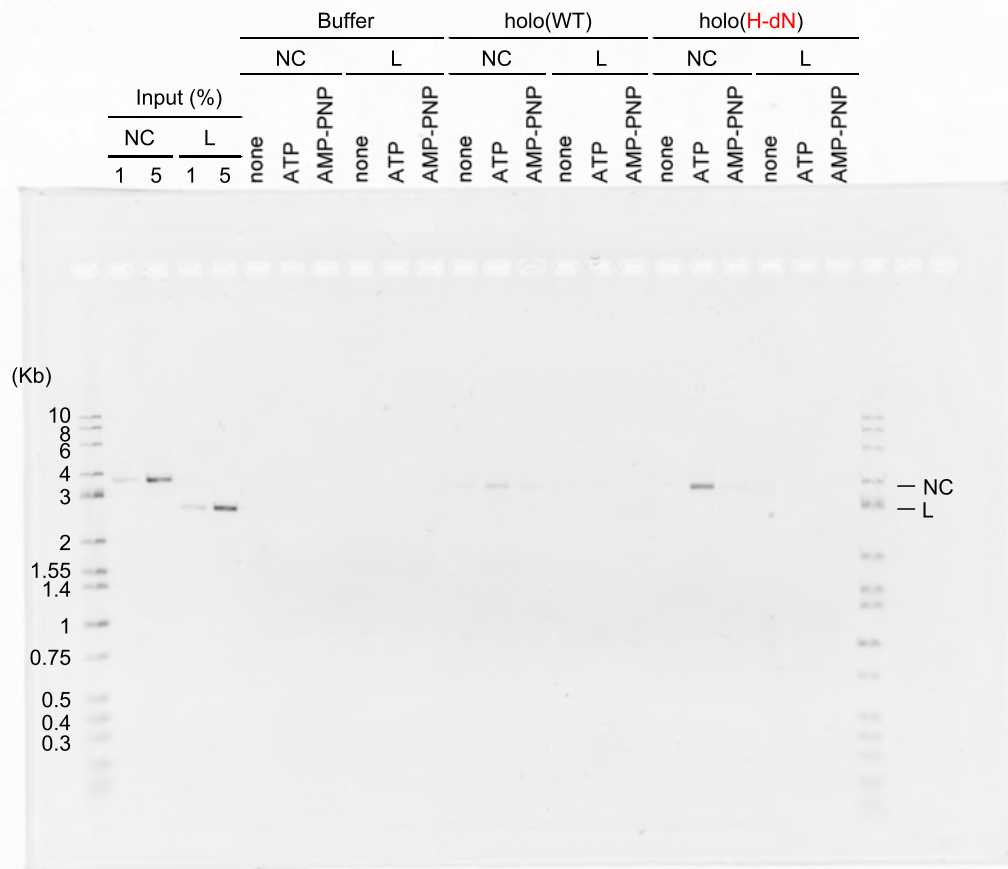

Supplement: Figure 4—source data 1. [file elife-84694-fig4-data1.zip › Figure 4-source data 1.pdf]

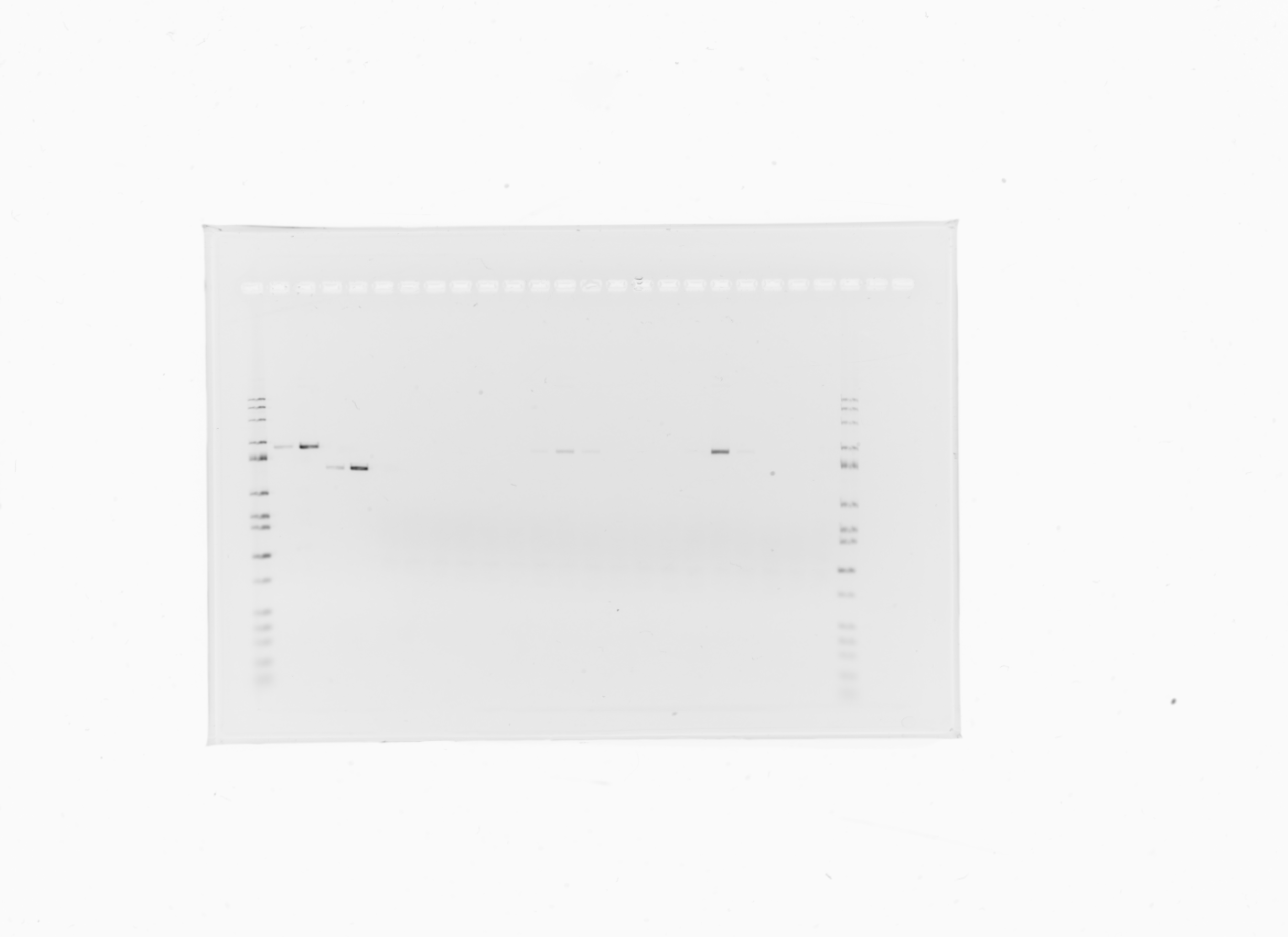

Supplement: Figure 4—source data 1. [file elife-84694-fig4-data1.zip › Figure 4-source data 1.tif]

Figure 4-source data 2  
Figure 4B

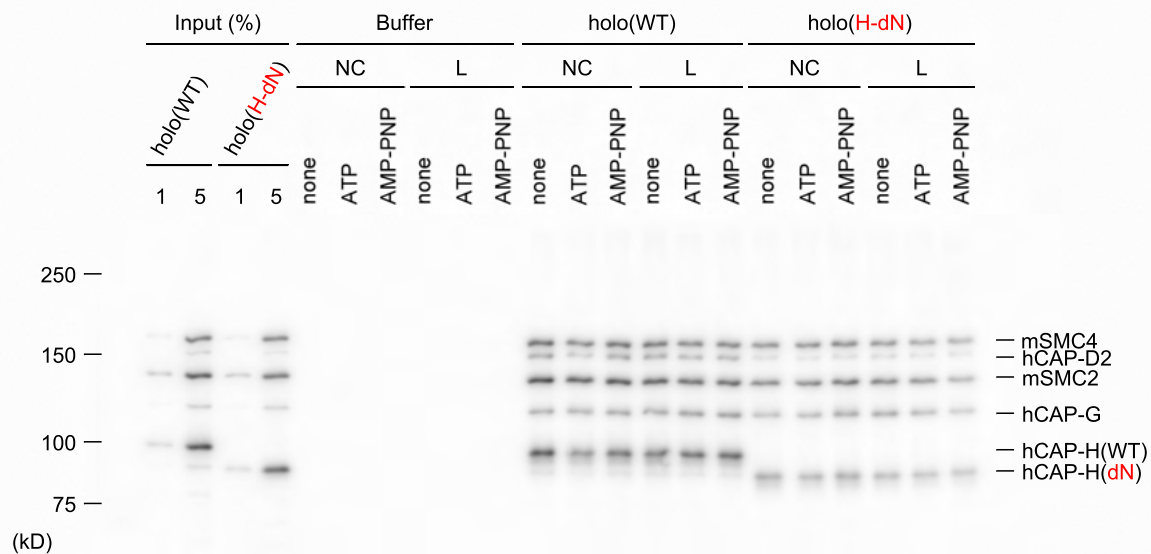

Supplement: Figure 4—source data 2. [file elife-84694-fig4-data2.zip › Figure 4-source data 2.pdf]

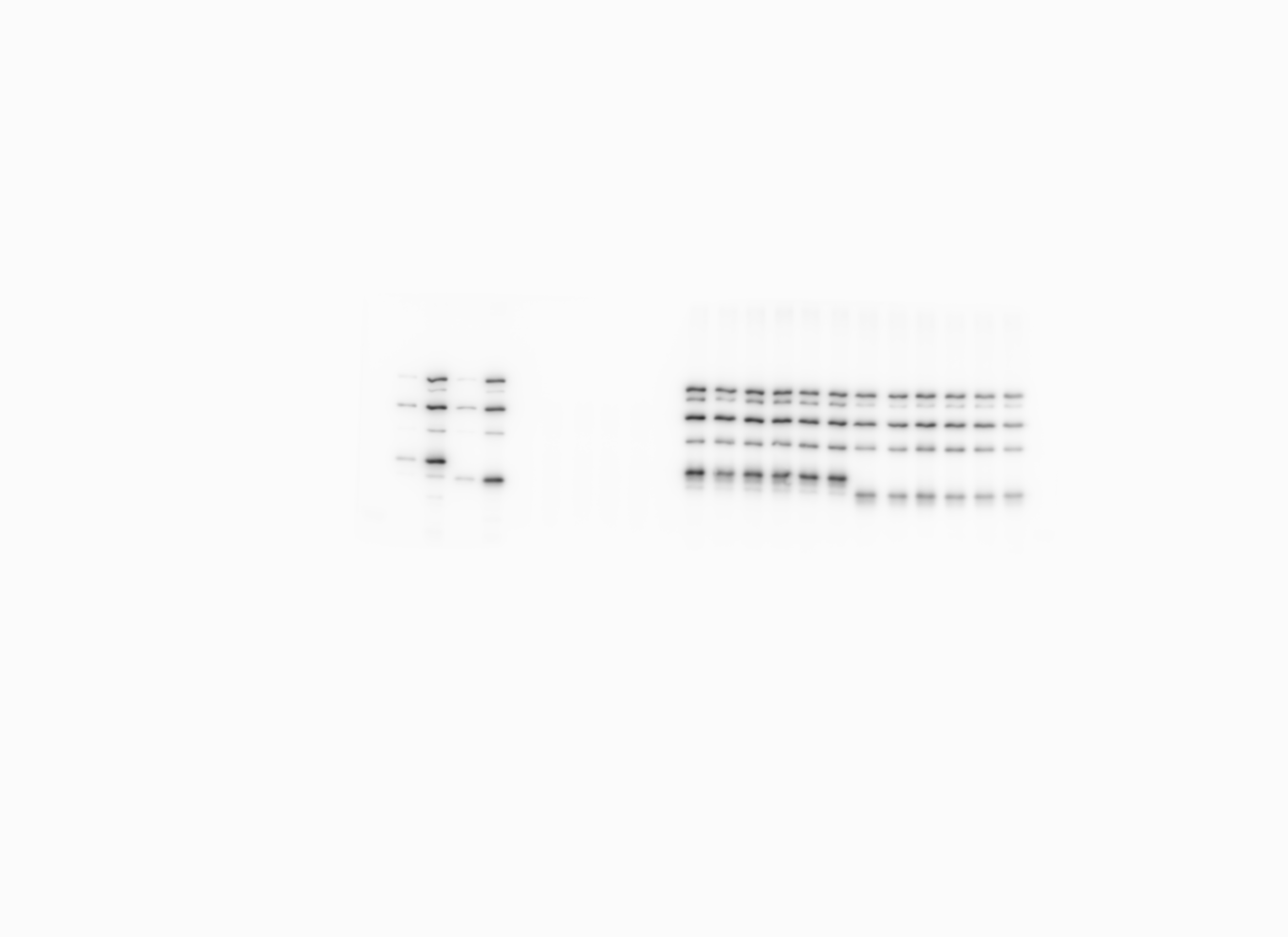

Supplement: Figure 4—source data 2. [file elife-84694-fig4-data2.zip › Figure 4-source data 2.tif]
